# Circadian System Development and Plasticity

**DOI:** 10.1155/2014/436760

**Published:** 2014-06-16

**Authors:** Martin Fredensborg Rath, Yoav Gothilf, Mario Guido, Estela Muñoz

**Affiliations:** ^1^Department of Neuroscience and Pharmacology, Faculty of Health and Medical Sciences, University of Copenhagen, Rigshospitalet 6102, Blegdamsvej 9, 2100 Copenhagen, Denmark; ^2^Department of Neurobiology, The George S. Wise Faculty of Life Sciences and Sagol School of Neuroscience, Tel-Aviv University, Sherman Building, Room 405, 69978 Tel Aviv, Israel; ^3^CIQUIBIC (CONICET), Departamento de Química Biológica, Facultad de Ciencias Químicas, Universidad Nacional de Córdoba, Haya de la Torre s/n, Ciudad Universitaria, 5000 Córdoba, Argentina; ^4^Laboratorio de Cronobiología, IHEM-CONICET, ANPCyT, Facultad de Ciencias Médicas, UNCuyo, CC 56, Parque Gral. San Martín, 5500 Mendoza, Argentina

Circadian clocks drive 24 h oscillations in physiology and behavior from algae to humans, using light/dark cycles as the main synchronizing input. The questions of where, when, and how these rhythms take place have inspired amazing advances in the field of circadian biology, resulting in the discovery of the major players that compose, regulate, and fine-tune a precise molecular clock mechanism. Nevertheless, how the whole circadian system is developed and what allows this system to be plastic and adaptive, along with precision, are questions that are still unclear. It is therefore of special interest to continue the efforts to understand the cellular, molecular, and genetic mechanisms behind the ontogeny and plasticity of circadian clocks. This special issue includes ten original articles and reviews. These contributions address different developmental and dynamic aspects of the circadian timing system from molecular and cellular mechanisms to synchronization of physiology and behavior, in diverse models such as zebrafish*, Xenopus laevis*, chicken, rodents, and primates, including humans. This diversity of species clearly reflects the evolutionary conservation of basic circadian mechanisms.

Starting with zebrafish, Z. Ben-Moshe et al. reviewed the group contributions and general knowledge about the maturation of the circadian clock in the fish pineal gland, finishing the manuscript with an open-ended question related to the influence of this and other peripheral oscillators in the physiology and behavior of the intact animal. They propose that the light-induced onset of the zebrafish pineal gland circadian clock is dependent upon entrainment of asynchronous cellular oscillators by light. Investigation and analysis of the light-regulated transcriptome and miRNome, among other approaches, suggest a complex mechanism with some similarities with those present in peripheral clocks.

The advantages of the zebrafish as a model species in the field of chronobiology are further emphasized by the study of K. Lahiri et al. These authors developed a reporter transgenic line which expresses luciferase under the control of the* Per1b* promoter. This reporter line and other molecular markers were then used to demonstrate that the zebrafish clock can be entrained by temperature cycles, as well as by light/dark cycles. They further show shifts in the developmental maturation of the response to temperature, which is an important step towards understanding the mechanism of temperature entrainment, and possibly temperature compensation. Future work is necessary to confront the mechanisms of entrainment induced by both* zeitgebers*, temperature and light.

M. N. de C. M. Moraes et al. investigated the effect of *α*-MSH and the nocturnal signal melatonin, on the expression of the clock gene* Per1* and nonvisual photopigments (melanopsins:* Opn4x* and* Opn4m*) in photosensitive melanophores of* Xenopus laevis*. It is known that light and *α*-MSH exert a dispersing effect on pigment granule distribution while melatonin facilitates their aggregation. Remarkably,* Per1* showed temporal oscillations regardless of the presence of either *α*-MSH or melatonin. On the contrary, the expression of* Opn4x* and* Opn4m* was differentially affected by these hormones: *α*-MSH increased melanopsin expression while melatonin effects depended on the time when it was applied, dramatically abolishing their temporal oscillations and decreasing their expression levels during the light phase.

Using the chicken as a model, N. M. Díaz et al. provide evidence that during early development, retinal melanopsinergic horizontal cells and ganglion cells express a number of core clock genes as well as the melatonin rhythm-generating enzyme AANAT. Their findings indicate that these circadian molecular features comprise a characteristic of melanopsinergic neurons of the developing inner retina which facilitates the development of the retinal circadian timing system.

D. S. Pereira et al. used the* Per3* knockout mouse to analyze the role of the clock gene* Per3* in the circadian rhythm of behavioral responsiveness. Their data show that upon transition to long days synchronization to the new light regime is delayed in the knockout mice, thus suggesting that* Per3 *is required for efficient circadian synchronization to the changing environment.

K. Rohde et al. present their current understanding of the role of homeobox genes, not only as phenotype determinants in the rodent pineal gland but also as regulators of adult pineal gland function, specifically of the melatonin rhythm generation. They introduced a new working model with the transcriptional factor CRX facilitating tissue specificity and daily changes in* Aanat* expression, the gene that encodes the rate-limiting enzyme of melatonin synthesis.

In the rat SCN, J. Alamilla et al. used perforated or whole cell patch clamp techniques to investigate the temporal (day versus night) and regional (dorsal versus ventral) GABAergic neurotransmission. Their results suggest an excitatory role for GABA in the SCN and further demonstrate both daily and topographic modulations of GABAergic postsynaptic currents in the central clock.

Liver is one clear example of a peripheral oscillator in which other synchronizing signals besides the light/dark cycle, such as food availability or daytime restricted feeding (DRF), are able to adjust daily rhythms. D. D. Ita-Pérez et al. show that DRF influences the daily variations of hepatic mRNA levels, stability, and enzymatic activity of GABA transaminase (GABA-T), thus suggesting that the liver GABAergic system responds to the metabolic challenges imposed by DRF and denoting the presence of a food-entrained oscillator.

In a study on primates, R. C. G. J. Engelberth et al. evaluated the influence of senescence on the relative neuronal and glial SCN populations in female marmosets. Neuronal loss and astrocyte increase are reported in the aged marmosets. The authors speculate that these changes might be related with rhythmic pattern variations in the aging central clock.

Finally, in a study on human material, M. Møller et al. show the presence of classical neuropeptidergic NPY-immunoreactive nerve fibers in fetal (around 4th month of gestation) and adult pineal gland, which are likely to modulate pineal function. According to the location in both the pineal stalk and pineal follicles, this NPYergic innervation might represent an independent nonsympathetic central regulatory pathway. The authors discuss and present possible origins for these fibers and the significance of their studies. Further investigations are required to understand NPY roles in pineal gland ontogeny and function.

